# Resilience among male laryngeal cancer patients treated with total laryngectomy

**DOI:** 10.1192/j.eurpsy.2025.1774

**Published:** 2025-08-26

**Authors:** L. S. Chaibi, A. Amri, O. Feki, M. Cheour, S. Hallit, F. Fekih-Romdhane

**Affiliations:** 1 Department of Psychiatry, Razi Hospital, Mannouba; 2 Head and Neck Carcinologic Surgery, Salah Azaiez Institute, Tunis; 3 Department of Psychiatry, University of Mannouba, Mannouba, Tunisia; 4 School of Medicine and Medical Sciences, Holy Spirit University of Kaslik, Jounieh, Lebanon

## Abstract

**Introduction:**

Laryngeal cancer patients who undergo total laryngectomy (TL) face significant physical, emotional, and psychological challenges. This group of patients often struggle with feelings of social isolation, depression, and anxiety, as the surgery alters both their appearance and daily interactions. Despite these difficulties, patients can experience remarkable resilience in adapting to life post-surgery, with important individual differences though. Understanding factors associated with resilience among laryngeal cancer patients who had TL is crucial for improving mental health interventions and enhancing rehabilitation efforts.

**Objectives:**

The objective of this study was to examine the resilience of patients who had TL for laryngeal cancer and identify the associated factors.

**Methods:**

We carried-out a descriptive cross-sectional study including 30 patients treated for laryngeal cancer with TL in the Head and Neck Carcinologic Surgery Department at Salah Azaiez Institute.The operations took place during the period 2019-2022.All patients completed the Arabic versions of the Brief Resilience Scale (BRS) and the Arabic Multidimensional Scale of Perceived Social Support (MSPSS).

**Results:**

The study involved 30 male participants with a mean age of 62 years (±10 years).The mean BRS score was 17.6 ± 3.6. Patients from urban areas had lower BRS scores (P=0.005). Additionally, a higher level of education, particularly secondary or higher, was associated with lower BRS scores (P=0.001). Similarly, a higher socio-economic status correlated with lower BRS scores (P=0.02). Furthermore, greater resilience was significantly associated with higher perceived social support (P=0.001).

**Conclusions:**

This study reveals that patients who have undergone total laryngectomy may experience varying levels of resilience based on socio-demographic factors and perceived social support. Higher social support is linked to greater resilience, emphasizing the need for robust support systems to aid in recovery. Further research is needed to refine support strategies for these patients.

**Disclosure of Interest:**

None Declared

